# First Evidence of Carp Edema Virus Infection of Koi *Cyprinus carpio* in Chiang Mai Province, Thailand

**DOI:** 10.3390/v12121400

**Published:** 2020-12-06

**Authors:** Surachai Pikulkaew, Khathawat Phatwan, Wijit Banlunara, Montira Intanon, John K. Bernard

**Affiliations:** 1 Department of Food Animal Clinic, Faculty of Veterinary Medicine, Chiang Mai University, Chiang Mai 50100, Thailand; 2 Research Center of Producing and Development of Products and Innovations for Animal Health and Production, Faculty of Veterinary Medicine, Chiang Mai University, Chiang Mai 50100, Thailand; montira.intanon@gmail.com; 3 Veterinary Diagnostic Laboratory, Faculty of Veterinary Medicine, Chiang Mai University, Chiang Mai 50100, Thailand; khathawat.ph@cmu.ac.th; 4 Department of Pathology, Faculty of Veterinary Science, Chulalongkorn University, Bangkok 10330, Thailand; wijit.k@chula.ac.th; 5 Department of Veterinary Biosciences and Public Health, Faculty of Veterinary Medicine, Chiang Mai University, Chiang Mai 50100, Thailand; 6 Department of Animal and Dairy Science, The University of Georgia, Tifton, GA 31793-5766, USA; jbernard@uga.edu

**Keywords:** carp edema virus, koi sleepy disease, koi *Cyprinus carpio*, gill, Chiang Mai

## Abstract

The presence of carp edema virus (CEV) was confirmed in imported ornamental koi in Chiang Mai province, Thailand. The koi showed lethargy, loss of swimming activity, were lying at the bottom of the pond, and gasping at the water’s surface. Some clinical signs such as skin hemorrhages and ulcers, swelling of the primary gill lamella, and necrosis of gill tissue, presented. Clinical examination showed co-infection by opportunistic pathogens including *Dactylogyrus* sp., *Gyrodactylus* sp. and *Saprolegnia* sp. on the skin and gills. Histopathologically, the gill of infected fish showed severe necrosis of epithelial cells and infiltrating of eosinophilic granular cells. Electron microscope examination detected few numbers of virions were present in the cytoplasm of gill tissue which showed an electron dense core with surface membranes worn by surface globular units. Molecular detection of CEV DNA from gill samples of fish was performed by polymerase chain reaction (PCR) and confirmed by nested-PCR. Phylogenetic analyses revealed that CEV isolate had 99.8% homology with the CEV isolated from South Korea (KY946715) and Germany (KY550420), and was assigned to genogroup IIa. In conclusion, this report confirmed the presence of CEV infection of koi *Cyprinus carpio* in Chiang Mai province, Thailand using pathological and molecular approaches.

## 1. Introduction

The cyprinid fish (family *Cyprinidae*), such as common (*Cyprinus carpio* L.) and koi (*Cyprinus carpio* koi), is one the most important freshwater fish groups in the world. The main common carp-producing countries for human consumption are found in Europe, South America, and Asia [1]. In addition, koi—a color variety of common carp—is a popular ornamental fish commonly cultured as a pet due to their beautiful color variations and body shape. Consequently, commercial aquaculture production of koi has developed as a major source of income for the pet industry and plays an increasing role in meeting worldwide demand for ornamental fish hobbyists [2]. Koi has been bred in Thailand since 1960, but are also imported into Thailand from Asian countries, especially from Japan. However, the international trade of koi transfers large numbers of live fish between different places, creating the potential for transmission of various pathogens, especially viruses, which often cause major damage to the aquaculture industry [3]. 

In recent decades, there have been many reports on important viral diseases affecting cyprinid fish. The major epizootics virus are usually caused by members of the *Alloherpesviridae* family: herpesviral haematopoietic necrosis (HVHN) caused by cyprinid herpesviruses 2 (CyHV-2) [4] and koi herpesvirus diseases (KHVD) caused by cyprinid herpesviruses 3 (CyHV-3) [5,6,7]. Spring viremia of carp (SVC), a fish rhabdovirus, is one of the most important virus affecting carp listed by the OIE (OIE, 2019) [8] as a notifiable animal disease [9]. Recently, carp edema virus (CEV) was identified as another virus that infects cyprinid fish, especially common carp and koi, which also may have the potential to cause severe economic losses in the carp aquaculture industry.

Carp edema virus disease, also known as koi sleepy disease (KSD), was first identified in the 1970s in Japan where it caused outbreaks of disease frequently resulting in high mortality among juvenile cyprinid fish, especially ornamental koi [10]. CEV is a double-stranded DNA virus belonging to the *Poxviridae* family, and is the etiology of KSD [11,12]. KSD has been reported world-wide including East-Central Europe [13,14,15,16], USA [17,18], India [19], Republic of Korea [20], and most recently in China, Czech, and Slovak [21,22]. The temperature where clinical signs of KSD occur ranges from 15 °C to 25 °C [15]. Significant presence of the disease results in high mortality rates up to 80–100% in juvenile koi, especially in association with stressful management conditions [23]. The typical abnormal behavior of infected adult fish is to lie on the bottom of the pond for long periods, which has led to the name KSD [15]. Moreover, common clinical signs of infected fish are lethargy, anorexia, excessive mucus, skin hemorrhages with edema of the tissues, skin ulceration around the mouth and base of the fins, enophthalmos, and pale swollen gills [10,23]. Significantly, pathological lesions on vital organs such as gill hyperplasia and gill necrosis occur, leading to high mortality with dyspnea and hypoxia condition in infected fish [23,24].

A presumptive diagnosis of KSD includes observation of morbidity and mortality rates and common clinical signs. However, because of the similarities of pathognomonic signs of viruses affecting in koi such as koi herpesvirus (KHV) and SVC, specific diagnostic procedures are required. Various CEV diagnostic assays have been established based on genetic detection of the virus by PCR [25,26]. Importantly, there have been no previous reports of KSD in Thailand until this date.

The purpose of this report is to provide data on the first evidence of carp edema virus infection in the northern part of Thailand using standard veterinary diagnostic examination and molecular techniques. This information is critical for consideration of CEV diagnosis and control programs in Chiang Mai province and throughout Thailand.

## 2. Materials and Methods 

### 2.1. Disease History and Clinical Examination

In October 2019, mass moribund juvenile *Cyprinus carpio* koi were reported in a hobbyist koi pond. The pond was located in San Kamphaeng (18°44′43″ N, 99°7′13″ E), Chiang Mai province. Based on owner evidence, 300 newly imported koi fish from Japan had been added to the concrete pond with a capacity of about 360,000 L. The stocking density was 1 fish per 1200 L. Water quality parameters (temperature, pH, total ammonia and nitrite) were weekly evaluated using commercial test kits. Clinical signs observation and water quality sampling were recorded on-site. Water temperature and pH were measured by dissolved oxygen (Model Y550A, YSI Incorporated, Yellow Springs, OH, USA) and pH (CyberScan 500, Eutech Instruments Pte Ltd., Ayer Rajah Crescent, Singapore) meters, respectively. Measurement of total ammonia nitrogen level in the water samples was analyzed according to the method of Grasshoff, Kremling, and Ehrhardt (1983) [27], and unionized ammonia (NH_3_) concentration was determined according to the method of Emerson, Russo, Lund, and Thurston (1975) [28].

The moribund fish were packed in oxygenated, water-filled plastic bags and delivered to the Aquatic Animal Medicine Laboratory of the Faculty of Veterinary Medicine, Chiang Mai University, Chiang Mai province, Thailand for veterinary diagnostic examination and treatment advice. According to the clinical standard examination, each fish was submitted for parasitological analysis of mucus on the skin and fin, as well as a gill scraping. Samples were examined with a light microscopy. Necropsy was performed under sterile conditions. Bacterial cultures from spleen and head kidney tissues were cultured aseptically and streaked on blood agar and incubated at 25 °C for 48 h.

### 2.2. Histopathological Study

Gill, spleen, and liver tissues were placed in 10% buffered formalin and allowed to fix for 24 h. Fixed tissues were routinely processed for histopathology in paraffin embedding, and 4-μm thickness tissue sections were stained with hematoxylin and eosin (H&E). 

### 2.3. Electron Microscopic Study

The formalin-fixed paraffin embedded block of gill was cut and deparaffinized in xylene, refixed with 0.1 glutaraldehyde, and then immersed in 0.1 M phosphate buffer (PB). Tissues were refixed in 2% glutaraldehyde in PB, post-fixed in 1% osmium tetroxide and epoxy-resin embedded. Ultrathin sections were double-stained with uranyl acetate and lead acetate and examined using a transmission electron microscope (JEM1400, JEOL Ltd., Tokyo, Japan).

### 2.4. Primers and PCR Amplification of CEV

DNA was extracted from the gills of three affected koi and one healthy koi sample using Nucleospin^®^ Tissue (Machery-Nagel GmbH, Dauren, Germany), as suggested by the manufacturer. Briefly, gills were incubated at 56 °C for 3 h with Proteinase K, followed by the addition of a lysis buffer, incubated at 70 °C for 10 min, and then treated with 96% ethanol for DNA precipitation. The mixture was then centrifuged at 11,000× *g* for 1 min. The supernatant was removed and the pellet was washed with washing buffer. The DNA pellet was dissolved using an elution buffer (5 mM Tris/HCl, pH 8.5). DNA concentration and purity were determined by DU^®^730 UV/Vis spectrophotometer (Beckman Coulter Life Sciences, Indianapolis, IN, USA), and the samples were stored at −80 °C until further analysis.

The DNA sample was primarily screened for KHV by standard PCR using 2 different methods [5,6] (primers are listed in Table 1). The reactions were carried out in 50 µL reaction, containing 25 µL of 1× KOD OneTM PCR Master Mix (TOYOBO CO., LTD., Osaka, Japan), 3 µL of each primer (10 µM), 2 µL of DNA template, and PCR grade water up to a final volume of 50 µL. The amplification cycles were denaturation at 98 °C for 10 s, annealing at 55 °C for 5 s, and extension at 68 °C for 1 s.

For CEV detection, the sample was tested using a conventional nested PCR protocol (according to Matras et al. 2017 [12]). The first PCR was carried out using the same protocol described above, with primers CEV-For-B and CEV-Rev-J (Table 1). The nested PCR was performed with oligonucleotide primers CEV-For-B Internal and CEV-Rev-J Internal (Table 1), and the amplified products obtained from the first reaction were used as a DNA template, and the PCR conditions were the same.

Five µL of amplified PCR products were detected by electrophoresis (Major science, Saratoga, CA, USA) on a 1.5% (*w/v*) agarose gel containing 0.05 μL/mL of RedSafe^TM^ (iNtRON Biotechnology Inc., Seongnam-Si, Republic of Korea) in 1× tris-acetate-ethylene diamine tetraacetic acid buffer (pH 8.0) and 100 bp ladder RTU (GeneDireX, Inc., Miaoli County, Taiwan). The gel images were captured using the image scanner GelMax^TM^ Imager (UVP LLC., Upland, CA, USA).

### 2.5. DNA Sequence and Analysis of Sequence Data

The PCR products were purified using MagExtractor-PCR & Gel Clean up- (TOYOBO CO., LTD., Osaka, Japan) as described in the manufacturer’s instructions. Purified PCR amplification products were partially sequenced using primers at the Genomic Division, Macrogen Inc., Republic of Korea, and by ABI PRISM.

The 478-bp partial 4a gene sequence obtained was analyzed by Clustal W for multiple alignments [29]. The sequence of CEV was compared and checked by using the BLAST program available on the National Center for Biotechnology Information database for species confirmation and collecting of essential sequences for phylogenetic analysis. The phylogenetic tree was constructed using the MEGA-X software version 10.1.8 [30]. A neighbour-joining tree was created using a maximum composite likelihood model, and the robustness of the tree was tested using 1000 bootstrap replicates.

## 3. Results

### 3.1. Disease History and Clinical Examination

**Figure 1:**
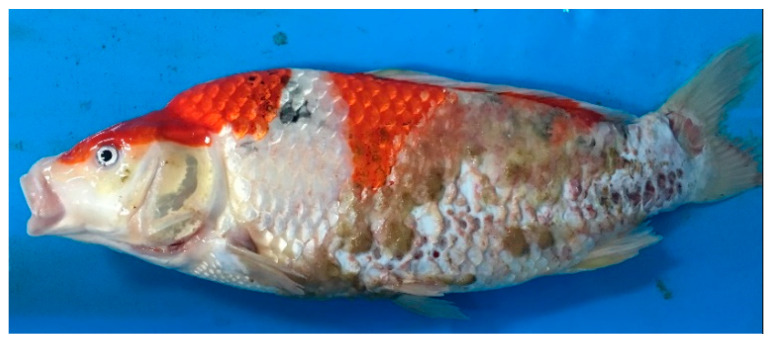
Gross examination of carp edema virus-infected *Cyprinus carpio* koi showing slightly sunken eyes and erosive and ulcerative skin with cotton wool-like.

**Figure 2:**
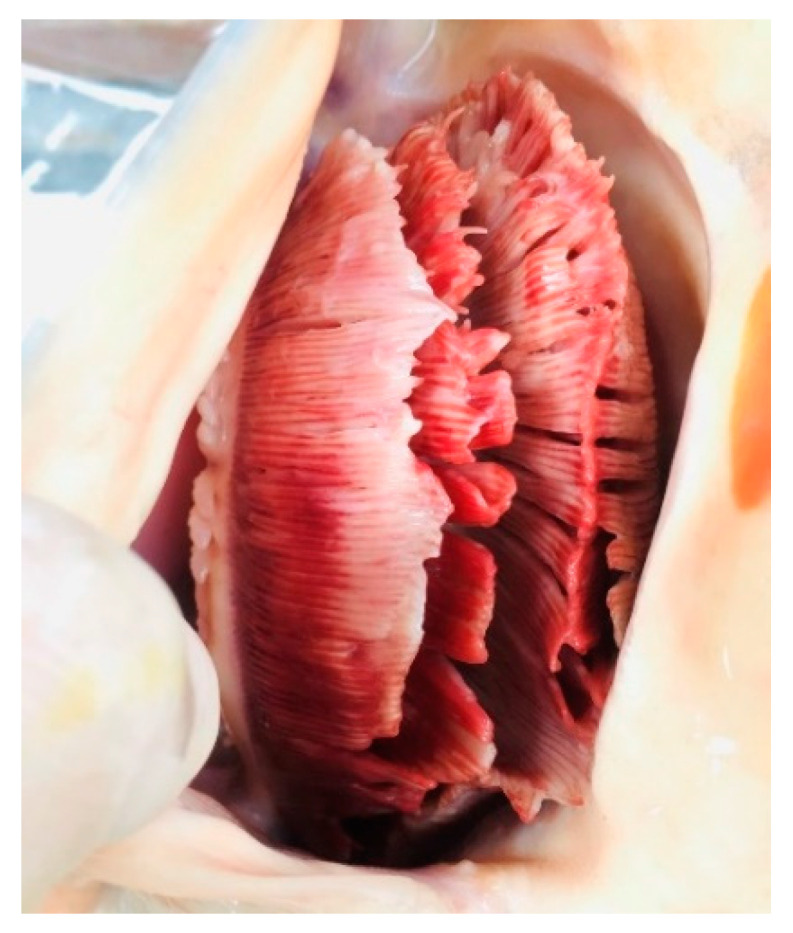
The gill of carp edema virus-infected fish (*Cyprinus carpio* koi) showing severe swelling of the primary filaments and necrosis of gill tissue.

Regarding the clinical symptoms, some affected koi were gathering near the aerated areas or sides of the pond, lying at the bottom of the pond, or gasping at the surface of water. The affected koi also displayed an abnormal behavior, including unresponsiveness, lethargy, and loss of swimming activity. Some fish were observed floating upside down or struggling to maintain a normal position. Additional clinical signs observed in some fish included skin hemorrhages and ulcers, body swelling, and cotton wool-like growths with grey-green color on the skin (Figure 1), sunken or cloudy eyes, swelling of the primary gill lamella, and necrosis of gill tissue (Figure 2). The number of fish exhibiting these signs increased daily. These abnormal observations started one week after rearing of koi with approximately 10% mortality and 70% morbidity on the day of the site visit. During the examination period, water quality was: 24–25 °C, 7.4 pH, 0.1 ppm total ammonia nitrogen level, and 0.02 ppm NH_3._ All parameters were within appropriate ranges for freshwater cyprinid fish. After 3 weeks of rearing, koi start to recover and the cumulative mortality rate in this case was approximately 20%

Microscopic examination revealed some infection by opportunistic organisms including *Dactylogyrus* sp. and *Gyrodactylus* sp. of skin, gill, and fins. The presence of fungal hyphae was detected by light microscopic observations and confirmed as *Saprolegnia* sp. (water molds). During necropsy, pale liver, liver congestion, and no content in gut were observed in some fish; in addition, one fish exhibited liver and kidney enlargement. No visible internal parasites were observed in any of the necropsied fish. After 48 h of incubation on blood agars plates, no bacterial growth was detected in spleen and head kidney issues. For supportive therapy, treatment of the pond with 0.3–0.5% sea salt was recommended to the owner. 

### 3.2. Histopathological and Electron Microscopic Study

**Figure 3:**
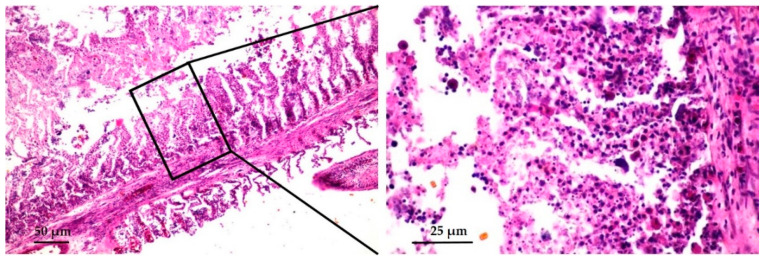
Histopathological studies of gills from CEV-affected koi. The gill filament showing severe necrosis of epithelial cells and infiltrating of eosinophilic granular cells. (hematoxylin and eosin staining, scale bar = 50 and 25 μm, respectively).

Histopathological analysis of gill tissue revealed fused lamellae with massive necrosis of epithelial cells showing massive pyknosis, karyolysis, and infiltration of many eosinophilic granular cells (Figure 3). The liver was swollen, with vacuolar degeneration of the hepatocytes and severe congestion in the sinusoids.

**Figure 4:**
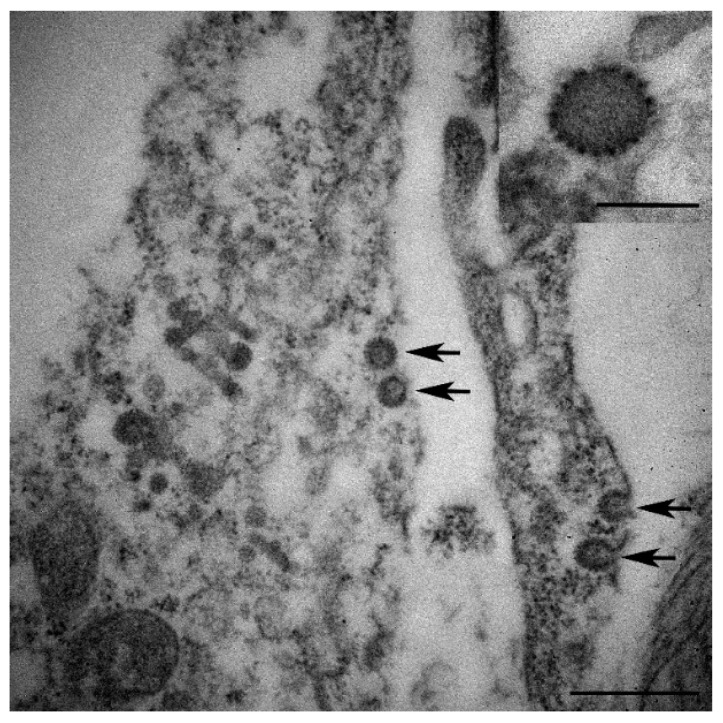
Electron microscopy of gills from CEV-affected koi. Few number of virions present in the cytoplasm (arrows). Scale bar = 500 nm. (Inset) Virion showing electron dense core with surface membrane worn by surface globular units. Scale bar = 200 nm.

Electron microscopy of the formalin-fixed paraffin embedded gill revealed a low number of virus particles (Figure 4). The particles had an electron dense core with a surface membrane that was worn by surface globular units. A lateral body was present beside the dense core. However, our results did not provide a clear photo, possibly due to the degradation of the tissue and the limitations of the microscope method from the formalin-fixed paraffin embedded tissue. 

### 3.3. CEV Confirmation by PCR 

**Figure 5:**
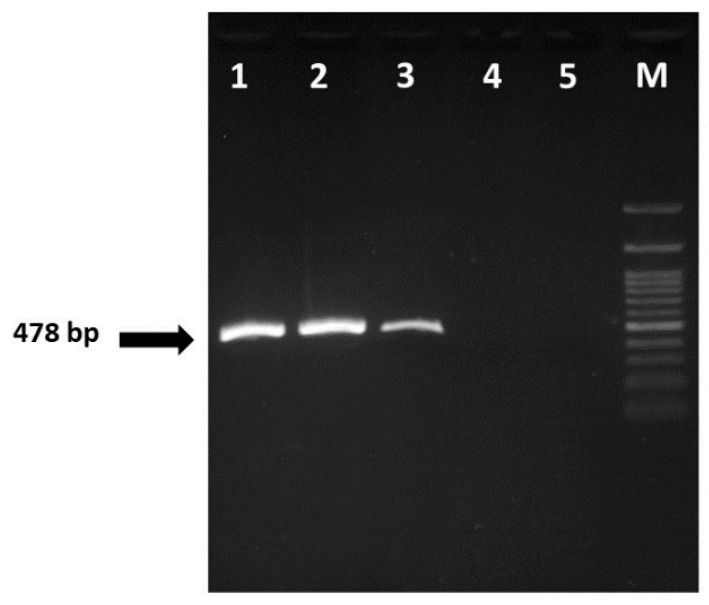
Detection of CEV DNA products (478 bp) by nested-PCR. Lane 1–3: gill samples of infected koi, lane 4: sample of healthy koi, lane 5: negative control with sterile water and lane M: 100 bp.

The results of PCR screening aiming to detect thymidine kinase gene or the enlarged 9/5 region of KHV were negative (data not shown) for all gill samples of affected koi. Nevertheless, all were positive in a single PCR round (with a 528 bp amplicon) and confirmed by nested PCR (478 bp amplicon size) (Figure 5). CEV was neither detected in healthy koi nor in the negative controls.

### 3.4. Sequence Analysis and Phylogenetic Study

**Figure 6:**
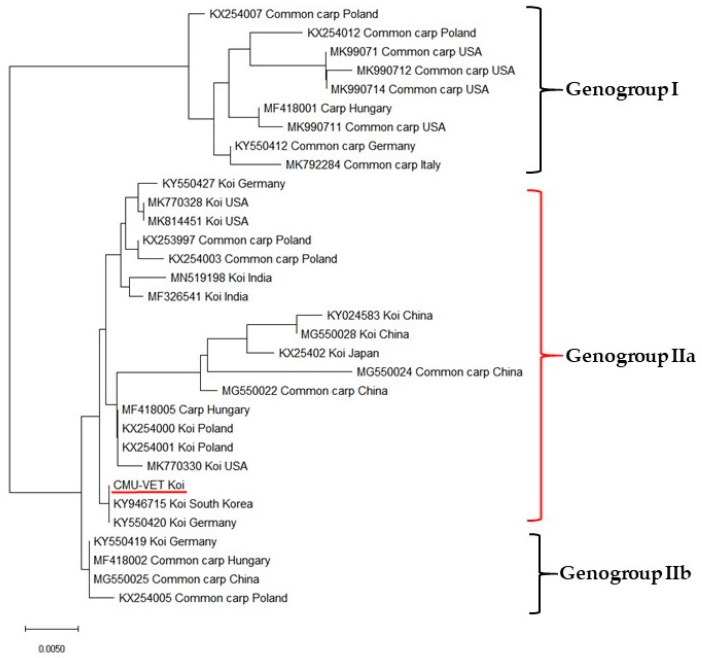
Phylogenetic tree based on a 478 bp partial 4a gene sequence of CEV and inferred by the neighbor-joining method using Mega X version 10.1. This analysis shows the sequences obtained from the infected koi from Chiang Mai, Thailand: CMU-VET koi, belong to genogroup IIa. The isolate fragment sequences shared 99.8 % nucleotide identity to the South Korea (KY946715) and Germany (KY550420). The scale bar represents substitutions per nucleotide site.

The 478-bp nested amplicon of the partial 4a gene of the isolate was purified and sequenced. The partial 4a gene sequences of CMU-VET (Chiang Mai University-Veterinary) koi shared 99.8% nucleotide identity with previous sequences obtained for South Korea (KY946715) and Germany (KY550420) by Blast alignment (Figure 6). Multiple sequence alignments and phylogenetic analysis were performed using partial 4a gene sequenced from 31 representative CEV and one sequence from this study. The difference of the nucleotide sequences of the partial 4a gene analysis allowed for identification of three genogroups of virus variants [12] in the phylogenetic tree, and the CEV sequence determined in this study included in genogroup IIa. 

## 4. Discussion

The results of this study confirmed the presence of CEV infection in koi imported from Asian countries to Thailand wholesalers of ornamental carp and transfer to koi keeper in Chiang Mai province. In the affected fish, some parasites and fungal pathogens were observed, but assumed to be caused by opportunistic infection. In addition, bacterial infection was proved to be negative in affected koi. Water quality parameters of the affected pond were within normal ranges, and therefore poor water quality was ruled out as cause of the mortalities. The results of the present clinical and laboratory examinations on the affected koi confirmed that virus infection was the etiology of the disease. Diagnosis was based on clinical signs, histopathology, detection of the viral particle via transmission electron microscope (TEM), and DNA identification via nested-PCR, which confirmed that gill lamellae epithelial cells of fish suffering from KSD were infected with CEV.

Water temperature is a significant factor in the outbreak of KSD. The first report of KSD in juvenile koi occurred during the rainy season at water temperatures between 15–25 °C [10]. In this report, the average water temperature when the clinical signs of disease occurred was between 24–25 °C during the Thailand rainy season. The cumulative mortality rate in this case was 20%, which is not consistent with previous reports [15,16,23]. Mortality and morbidity rates unusually decrease when temperatures increase to 28 °C [26], which may explain the lower mortality observed in this case. Nevertheless, some previous reports indicate that KSD occurs at lower temperatures. For example, an outbreak of KSD in common carp and koi was observed at 7–15 °C in Austria [13]. Various factors such as stress associated with handling, stocking, transportation, and water quality, especially water temperature, play a critical role in the outbreak of viral infection in aquatic animals [4,31,32]. To this regard, mortality has been reported to reach 80–100%, especially in juvenile koi when fish are transferred to another rearing ponds under stressful conditions [23]. In addition, our previous report of KHV infection in koi carp in Chiang Mai province, Thailand also occurred during the rainy season with stress conditions [7]. Thus, management practices to prevent an outbreak of this diseases include avoiding stressful conditions during periods of temperature fluctuation, and checking water quality routinely, combined with essential clinical examination of any suspect fish. 

The most common behavioral abnormality of CEV infection in cyprinid fish is lying on the bottom of the ponds. Other clinical signs of infected fish include: lethargy, anorexia, swelling and necrotic gills, enophthalmos, and skin hemorrhages or skin ulceration [23,24,25,26]. Similar clinical signs were observed in the present episode. Unfortunately, some fish owners or breeders may transfer infected fish with asymptomatic shedding without a confirmative diagnosis of disease. This would potentially spread CEV to other fish, and so therefore a veterinary approach is needed including the diagnosis of aquatic animal disease with precise and reliable tools. Moreover, clinical signs of CEV infection are similar to other cyprinid viral infections such as KHV or SVC [23,26], but the presence of spring viremia of carp virus was not ruled out in this outbreak.

It was interesting that many previous reports of CEV-positive fish were co-infected with bacterial pathogens, in particular infection with *Aeromonas* sp. and [12,13] and *Flavobacterium branchiophilum* [33]. In the present study, no bacterial growth from spleen and head kidney tissue was observed. A previous report demonstrated that high amount of opportunistic *Aeromonas* spp. were isolated from skin, but the samples collected from internal organs had no bacterial growth [15]. In addition, the present clinical examination of gill and skin from affected fish showed that monogeneans (*Dactylogyrus* sp. and *Gyrodactylus* sp.) were found commonly as opportunistic parasites. Secondary infection of various parasites were also reported in association with CEV infection such as *Gyrodactylus* sp. *Dactylogyrus* sp., *Trichodina* sp., *Ichthyobodo necator* and *Ichthyophthirius multifilis* [13,23,34]. We also found *Saprolegnia* sp. as an opportunistic infection and this was in agreement with Ademek et al., 2018 [16]. The high number of opportunistic infections suggest that the fish affected by the virus might be immunocompromised, which causes mortalities in fish farm [16]. Interestingly, some reports revealed coinfection associated with CyHV-3 and CEV in the Republic of Korea and China which caused mass mortality in koi [20,21]. Both viruses are double stranded DNA with similar clinical signs and can infect common carp and ornamental koi.

The mulberry-like envelope that is characteristic of the pox virus group has been confirmed in gill tissue [34]. According to TEM data on viral morphology of gill samples, the particles have an electron dense core with a surface membrane worn by surface globular units, indicating that the cause of the disease could be a poxvirus. In addition, the histopathological examination of affected fish exhibited pathological changes in the gill tissue including massive necrosis of epithelial cells and infiltrating of many eosinophilic granular cells. Acute tissue injury causes degranulation of the eosinophilic granule cells and increases pro-inflammatory responses of tissue [35]. These pathological changes were also observed in previous reports of fish affected by CEV infection [16,21]. Ouyang 2018 et al. [36] also reported swelling and congestion of gill lamellae of infected fish. The major function of gills and skin are involved in osmoregulation in fish [37]. These gross pathology and histopathological findings suggest a greater possibility for secondary infections by ectoparasites, bacteria, or fungus that would disrupt barrier function of the gills and skin, causing mortalities in infected fish in the present study.

For molecular epidemiology, previous phylogenetic study of the p4a gene showed two main lineages of CEV virus: lineage 1, usually found in koi, and lineage 2, usually isolated from common carps [38]. More recently, the classification of CEV was divided into three genetic genogroups including genogroup I, IIa and IIb [16,25]. Viruses from genogroup I have been reported in common carp [12,25] whereas CEV from genogroup IIa were mostly found in koi [11,12,25]. The sequence of our isolate (CMU-VET koi) has 99.8% homology with South Korea (KY946715) and Germany (KY550420) strains, which are assigned to genogroup IIa.

Overall, we concluded that the infected fish are the first evidence of CEV infection in koi in Chiang Mai province, Thailand. The results of this report confirmed the presence of CEV based on clinical signs, histopathology, transmission electron microscope, and nested-PCR from gill samples. The principles of biosecurity and appropriate husbandry must be applied by individual koi keepers, koi fish farmers, and koi wholesalers. Importantly, quarantine of imported cyprinid fish species should be performed with awareness of CEV. We propose including the CEV diagnosis with rapid and reliable method such as PCR into routine diagnostics of national and regional aquatic animals disease laboratories. A better understanding of the epidemiology of KSD is important, and the knowledge of disease surveillance will be helpful to prevent spreading the virus from koi to common carp aquaculture or to wild common carp. Last, but not least, further studies must be conducted to fill the information gaps in the treatment and prevention strategies of the disease in Thailand. 

## Figures and Tables

**Table 1:** List of oligonucleotide primer use in this study.

Primer	Primer Sequence (5′-3′)	Primer Length (bp)	Reference
KHV-TK-F	GGG TTA CCT GTA CGA G	16	Bercovier et al. (2005) [5]
KHV-TK-R	CAC CCA GTA GAT TAT GC	17	
KHV9/5F	GAC GAC GCC GGA GAC CTT GTG	21	Gilad et al. (2002) [6]
KHV9/5R	CAC AAG TTC AGT CTG TTC CTC AAC	24	
CEV-For-B	ATG GAG TAT CCA AAG TAC TTA G	22	Matras et al. (2017) [12]
CEV-Rev-J	CTC TTC ACT ATT GTG ACT TTG	21	
CEV-For-B Internal	GTT ATC AAT GAA ATT TGT GTA TTG	24	
CEV-Rev-J Internal	TAG CAA AGT ACT ACC TCA TCC	21	
